# Patient reported outcome measures concerning urinary incontinence after robot assisted radical prostatectomy: development and validation of an online prediction model using clinical parameters, lower urinary tract symptoms and surgical experience

**DOI:** 10.1007/s11701-020-01145-9

**Published:** 2020-09-15

**Authors:** Eelco R. P. Collette, Sjoerd O. Klaver, Birgit I. Lissenberg-Witte, Dies van den Ouden, Reindert J. A. van Moorselaar, André N. Vis

**Affiliations:** 1 Department of Urology, Maasstad Hospital, Rotterdam, The Netherlands; 2 Department of Urology, Amsterdam UMC, VU University Medical Center, room 4F27, De Boelelaan 1117, Postbus 7057, Amsterdam, 1081HV The Netherlands; 3 Department of Epidemiology and Data Science, Amsterdam UMC, VU University Medical Center, Amsterdam, The Netherlands

**Keywords:** Prostate cancer, Radical prostatectomy, Proms, Incontinence, Predictors, Prediction model

## Abstract

The prediction of post-prostatectomy incontinence (PPI) after robot-assisted radical prostatectomy (RARP) depends on multiple clinical, anatomical and surgical factors. There are only few risk formulas, tables or nomograms predicting PPI that may assist clinicians and their patients in adequate risk counseling on postoperative side-effects. Prospective data collection of 1814 patients who underwent RARP between 2009 and 2017 was done. Pre-operative parameters were age, body mass index (BMI), prostate volume, the American Society of Anesthesiologists (ASA) score, severity of Lower Urinary Tract Symptoms (LUTS), type of planned nerve-sparing surgery and surgical experience. The continence status was reported using Patient Reported Outcome Measurements (PROMs) using the validated pad-use questionnaire EPIC26. Continence was defined as either the use of zero pads or one safety pad. Multivariable logistic regression analysis was performed to identify predictors of PPI within one year after RARP. An online prediction tool was developed and validated. The median follow-up was 36 months (range 12–108). The response rate was high at 85.2%. A total of 85% (1537/1814) of patients was continent on follow-up. One-year continence rate was 80.1% (95% CI 78.3–81.9%) (1453/1814) and increased to 87.4% (95% CI 85.4–89.4%) after 5 years. On multivariable analysis, severity of LUTS (OR = 0.56 *p* = 0.004), higher age (OR = 0.73 *p* = 0.049), extend of nerve-sparing surgery (OR = 0.60 *p* = 0.001) and surgeon experience (OR = 1.48 *p* = 0.025) were significant independent predictors for PPI. The online prediction model performed well in predicting continence status with poor discrimination and good calibration. An intuitive online tool was developed to predict PPI after RARP that may assist clinicians and their patients in counseling of treatment.

**Electronic supplementary material:**

The online version of this article (10.1007/s11701-020-01145-9) contains supplementary material, which is available to authorized users.

## Introduction

With the increasing public awareness on prostate cancer and opportunistic screening methods, men are being diagnosed with localized prostate cancer at an earlier stage of disease. At present, prostate cancer is the most frequently diagnosed malignancy in men of 50 years and older. Radical prostatectomy (RP) is the most important surgical curative approach in prostate cancer patients. One of the negative consequences next to erectile Dysfunction of the surgical procedure is postoperative prostatectomy incontinence (PPI) [1]. PPI has a substantial negative effect on the overall health-relatively quality of life (HRQoL) and satisfaction of patients who underwent this surgical procedure [2].

In a systematic review of more than 8000 men who underwent robotic, laparoscopic or open RP, rates of PPI ranged from 4 to 31%.[2]. The literature concerning the predictive value of different pre-operative variables for PPI shows conflicting results. PPI is influenced by a wide set of pre-operative clinical variables, anatomic patient characteristics, per-operative surgical techniques, surgeon experience, as well as by the definition of urinary incontinence, the methodology to assess its impact and by the methods used to collect and report these data [2–5]. In these former studies on PPI, increased age and higher body mass index (BMI), higher comorbidity index, severity of lower urinary tract symptoms (LUTS) and larger prostate volumes were the most relevant pre-operative predictors of PPI after RARP [2].

There are, however, only few risk formulas, risk tables and nomograms predicting PPI after robot assisted radical prostatectomy (RARP) [6]. Existing models are hampered by low level of statistical power due to small sample size or by the absence of important predictive factors for PPI into the model such as surgical technique and surgical experience. By our knowledge, no online prediction model of PPI yet exists. We aimed to evaluate the weight of clinical, as well as different surgical variables to predict PPI after RARP. We developed and validated an online tool that may assist clinicians and their patients in adequate counseling of the risk for PPI after RARP.

## Patients and methods

### Patients

Data on PPI were collected from a longitudinal consecutive and institutional RARP database which was founded at the start of the robot program in 2009. Patients underwent RARP in a single center reference institute between January 2009 and October 2017. In this time period, a total of 2178 patients underwent RARP performed by four urological surgeons. All clinical, biochemical, pathological and radiological variables as well as follow-up data were prospectively collected in a comprehensive database. For the present study, patients were only included if the study follow-up time was at least 1 year.

### Defining urinary (in)continence

Patient reported outcome measures (PROMS) were used to assess PPI on postoperative follow-up. Urinary continence was defined as either the use of zero pads or the use of one safety pad according to the guidelines of the European Association of Urology (EAU) and using the EPIC26 questionnaire [1]. The use of one or more pads was defined as ‘postoperative urinary incontinence (PPI)’ according to the established literature [2, 4, 7].

Patients received the EPIC26 Questionnaire pre-operatively and at set time points after surgery. In the first postoperative year, questionnaires were handed over at three months and at 1 year. PROMS were performed up to ten years of follow-up. Handing over and sending the PROMS was performed by a dedicated nurse practitioner who also processed the data recorded on the paper forms into the database. If patients did not return the PROMS on first invitation, a repeated questionnaire was sent. When patients did not respond to the second invitation to fill in their PPI, they were called by telephone by the nurse practitioner and were asked on their urinary continence outcome. If patients could not be reached even after telephonic consulting, they were assumed lost to follow-up. Besides the EPIC questionnaire, patients also received the International Prostate Symptom Score (IPSS) questionnaire for evaluation of LUTS preoperatively as well as at three months and at one year postoperatively.

### Pre-operative parameters

At study inclusion, age, BMI (kg/m^2^), prostate volume (mL), and the American Society of Anesthesiologists (ASA) performance status were assessed. The severity of LUTS was assessed using the IPSS questionnaire.

### Per-operative parameters

The surgical procedure was assessed with respect to the extent of nerve-sparing surgery (i.e., both sided, one sided and no nerve-sparing surgery). The surgical experience was expressed as the surgically case numbers in groups of 100 patients. Definition of surgical experience is by author’s consensus and previously published work on surgical learning curve [8]. Furthermore, we defined a ‘novice’ surgeon as one having performed less than 100 surgical procedures, whereas a ‘senior’ surgeon performed over 100 RARP. One surgeon (SK) was marked ‘senior’ surgeon and performed over 80% (*n* = 1,469) of RARP and trained the other three ‘novice’ surgeons.

### Outcome measures

The primary outcome was the number of patients with PPI at 1 year (or vice versa, the number of patients who were ‘continent’ defined as having no PPI). Time to postoperative continence was defined as the time from surgery to the moment patients reported the use of zero pads or one safety pad.

Patients who underwent salvage radiotherapy on follow-up due to a local relapse of disease or in whom androgen receptor inhibitor medication or androgen deprivation therapy (ADT) was initiated due to recurrent (lymph-node) metastatic disease were censored at the time of the last continence questionnaire follow-up moment. In these patients, the outcome of the latest follow-up date of the evaluation of the urinary continence status was used in the analysis. Patients with bothersome PPI who underwent an AdVance male sling by AMS procedure or in whom an artificial urinary sphincter prosthesis placement was done were also censored at the latest follow-up. These patients were reported as being incontinent (having PPI) on the latest date of follow-up.

### Statistical analyses

All statistical tests were performed using SPSS® Statistics version 26.0 (IBM Corp., Armonk, NY, USA). Logistic regression models were used to assess the association between the pre-operative parameters and the primary outcome measure. Two multivariable logistic regression models were build. The first (large) model included all pre-operative parameters, irrespective of their influence on the primary outcome measure. The second (small) model was built with a backward selection procedure, in which one by one the least significant parameter was excluded from the model (*p* value for removal > 0.05). Receiver operative characteristics (ROC) curves with the predicted probabilities of both models were made and the area under the ROC curve (AUC) was computed, with corresponding 95% confidence intervals (CI). We performed tenfold internal cross-validation using R for Windows (version 3.6.1.). With the use of the regression coefficients of the small multivariable logistic regression model we built an online prediction model by means of the Evidencio platform (Evidencio Medical Decision Support, Haaksbergen, the Netherlands, Evidencio.com). The cumulative incidence of continence was estimated by a Kaplan–Meier curve. Continuous parameters were transformed to categorical variables using known clinical cut-offs or tertiles/quintiles, as the linearity assumption was not met.

## Results

The median follow-up time for the whole patient series was 36 months (range 12–108). The response rate for returning the EPIC26 questionnaire at one year of follow-up was 85.2% (1856 of 2178 patients). A total of 322 patients were not evaluable for analysis due to not returning the questionnaire, or because these patients were lost to follow-up. In total, 2.3% (42/1856) of patients returned their PROMs questionnaire, but were not assessable for analysis due to several other reasons (such as no answer to the questions, unreadable PROMS). This led to 1814 patients being evaluable for analysis. Pre-operative parameters of evaluated patients are listed in Table 1.

**Table 1:** Baseline pre-operative and per-operative patient characteristics: age, ASA score, body mass index (BMI), prostate volume, PSA-level, severity of lower urinary tract symptoms (LUTS), case number per surgeon and the extent of nerve-sparing surgery

Variable	Frequency
Age (years)	Median 66 years (range 40–79)
≤ 60	21.6% (392/1814)
61–64	19.9% (361/1814)
65–67	20.1% (364/1814)
68–70	19.1% (347/1814)
≥ 71	19.3% (350/1814)
ASA (score)	
1	32% (573/1814)
2	64% (1170/1814)
3	4% (71/1814)
BMI (index)	Median 26 (range 16–54)
Normal (< 25)	30.3% (550/1814)
Overweight (25–30)	56.3% (1021/1814)
Obese (> 30)	13.4% (243/1814)
Prostate volume (mL)	Median 54 mL (range 17–260)
≤ 50	43.0% (780/1814)
51–65	27.4% (497/1814)
> 65	29.6% (537 / 1,814)
Initial PSA (ug/l)	median 9.0 ng/mL (range 0.6–172.0)
Pre-operative LUTS (IPSS score)	Median 9 (range 0–35)
No or mild (0–7)	43.2% (784/1814)
Moderate (8–19)	43.2% (785/1814)
Severe (20–35)	13.5% (245/1814)
Case numbers per surgeon	
Surgeon 1 “senior”	81.0% (1469/1814)
Surgeon 2 “novice”	8.0% (146/1814)
Surgeon 3 “novice”	8.4% (153/1814)
Surgeon 4 “novice”	2.5% (46/1814)
Surgical caseload	
Cases by “novice” surgeon (1–100 cases)	11.0% (200/1814)
Cases by “senior” surgeon (> 100 cases)	89.0% (1614/1814)
Nerve sparing prostatectomy groups	
Group 1—Bilateral nerve sparing	54.6% (990/1814)
Group 2—Left or Right or Partial (one sided)	25.7% (466/1814)
Group 3—No nerve sparing	19.7% (358/1814)

*ASA* American Society of Anesthesiologists, *BMI* Body mass index, *LUTS* Lower urinary tract symptoms, *IPSS* International Prostate Symptom Score, *PSA* Prostate-specific antigen, *NSS* nerve sparing surgery

### Primary outcome of PPI

The one-year continence rate was 80.1% (1453/1814). On study follow-up, 85% (1537/1814) of patients was continent at 36 months of median follow-up. A cumulative incidence after five year follow-up of 87.4% (95% CI 85.4–89.4%) was reported.

### Univariate and multivariate analysis of PPI

The results of univariate regression analysis are listed in Table 2. All pre-operative variables were statistically significant associated with PPI after 1 year. Results of the multivariable logistic regression model is reported in Table 3. The backward selection procedure (large model) revealed that in our cohort, higher pre-operative IPSS, higher age, the extent of nerve sparing surgery and (lesser) surgeons experience were significant predictors of 1 year post-operative incontinence, while ASA, BMI and prostate volume were not. Table 3 also shows the results of the small prediction model after stepwise exclusion of the non-significant variables.

**Table 2:** Pre-operative and per-operative variables in association to post-operative continence at one year postoperatively. Univariate analysis

Pre- and per-operative variables	Continence (0 or 1 safety pad)	*p* value*
ASA—status 1	83.2% (477/573)	0.010
ASA—status 2	79.2% (927/1170)	
ASA—status 3	69.4% (49/71)	
BMI—normal < 25	80.6% (443/550)	0.039
BMI—overweight 25–30	81.3% (830/1021)	
BMI—obese > 30	74.1% (180/243)	
Prostate volume—≤ 50 mL	83.6% (652/780)	0.001
Prostate volume—51–65 mL	79.8% (397/497)	
Prostate volume—> 65 mL	75.0% (403/537)	
Age ≤ 60 years	84.1% (333/396)	0.001
Age 61–64 years	82.9% (300/263)	
Age 65–67 years	83.1% (304/366)	
Age 68–70 years	75.0% (261/348)	
Age ≥ 71 years	74.6% (262/351)	
LUTS—no or mild	83.5% (638/764)	0.002
LUTS—moderate	79.2% (609/769)	
LUTS—severe	73.6% (178/242)	
Nerve sparing group 1—bilateral NS	84.9% (841/990)	0.001
Nerve sparing group 2—left or right or partial (one sided)	74.4% (332/466)	
Nerve sparing group 3—non NS	74.3% (266/358)	
Novice surgeon 1–100 RARPs	72.5% (145/200)	0.004
Senior surgeon > 100 RARPs	81.0% (1307/1614)	

*ASA* American Society of Anesthesiologists, *BMI* Body mass index, *LUTS* Lower urinary tract symptoms, *IPSS* International Prostate Symptom Score, *NSS* nerve sparing surgery

*Pearson Chi-Square test

**Table 3:** Pre-operative and per operative variables in association to post-operative continence at one year postoperatively

Pre- and per-operative variables	Large model*		Small model*		
	OR (odds ratio)	*p* value	OR (odds ratio)	*p* value	95% CI
Nerve sparing group 1—bilateral NS	1	0.001	1	< 0.001	
Nerve sparing group 2—left or right or partial (one sided)	0.60		0.58		0.44–0.77
Nerve sparing group 3—non NS	0.62		0.58		0.43–0.80
LUTS—no or mild	1	0.010	1	0.004	
LUTS—moderate	0.75		0.75		0.58–0.97
LUTS—severe	0.59		0.56		0.40–0.80
Novice surgeon 1–100 RARPs	1	0.012	1	0.025	
Senior surgeon > 100 RARPs	1.6		1.5		1.05–2.1
Age ≤ 60 years	1	0.11	1	0.049	
Age 61–64 years	1.05		1.01		0.68–1.5
Age 65–67 years	1.1		1.04		0.70–1.5
Age 68–70 years	0.70		0.67		0.46–0.97
Age ≥ 71 years	0.79		0.73		0.50–1.1
ASA—status 1	1	0.22		n.s	
ASA—status 2	0.88				
ASA—status 3	0.60				
Prostate volume—≤ 50 mL	1	0.13		n.s	
Prostate volume—51–65 mL	0.91				
Prostate volume—> 65 mL	0.74				
BMI—normal < 25	1	0.11		n.s	
BMI—overweight 25–30	1.1				
BMI—obese > 30	0.77				

Multivariate analysis

*Logistic regression models were used to assess the association between the pre-operative parameters and the primary outcome measure. Two multivariable logistic regression models were build. The first (large) model included all pre-operative parameters, irrespective of their influence on the primary outcome measure. The second (small) model was built with a backward selection procedure, in which one by one the least significant parameter was excluded from the model (*p* value for removal > 0.05)

*ASA* American Society of Anesthesiologists, *BMI* Body mass index, *LUTS* Lower urinary tract symptoms, *IPSS* International Prostate Symptom Score, *NSS* nerve sparing surgery, *n.s.* non-significant

### ROC curve analysis and online prediction model

**Fig. 1:**
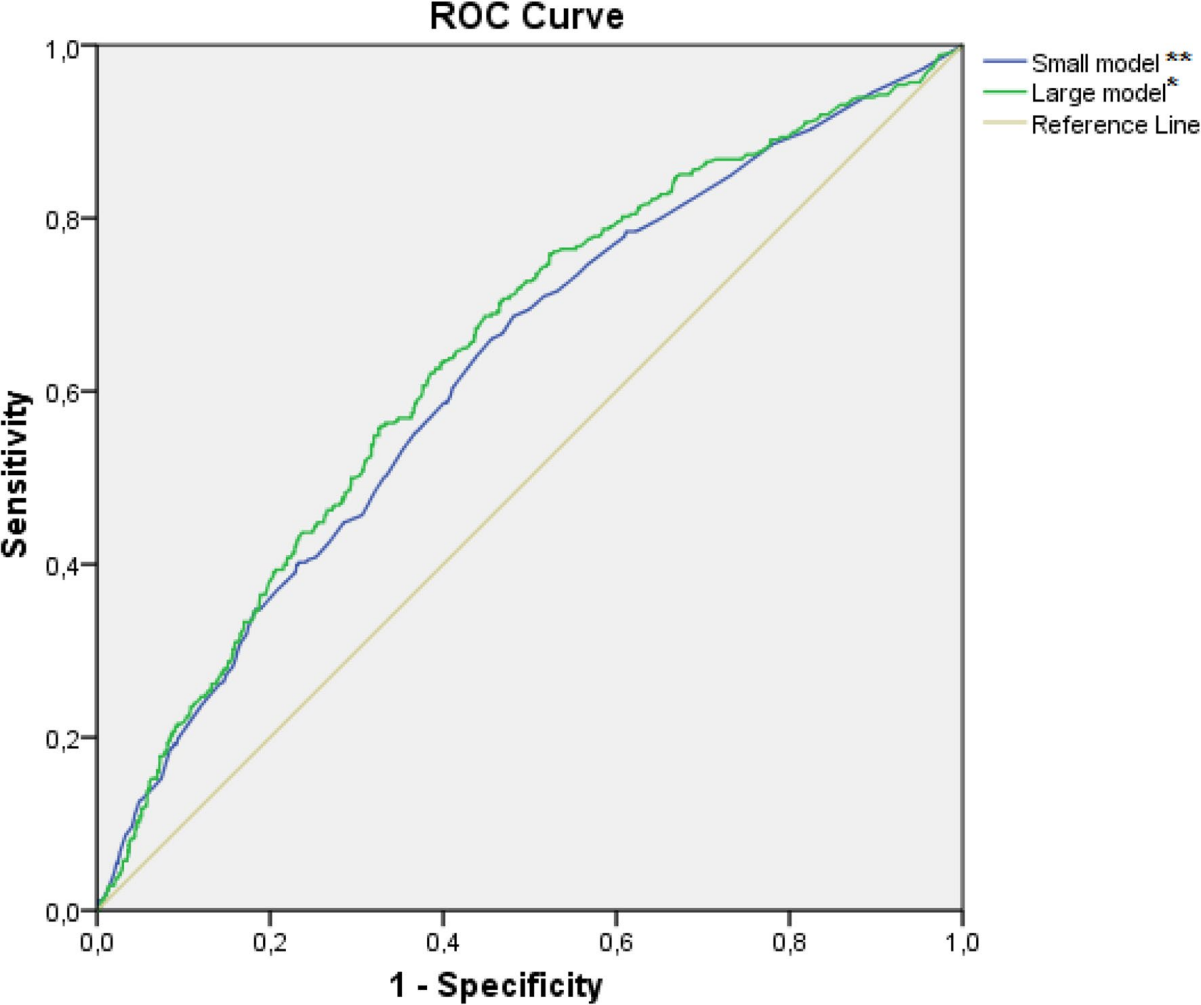
Receiver operative characteristics curve for large and small multivariable regression models. *Large model includes variables: NSS, LUTS, Age, surgical volume, BMI, ASA, Prostate volume, **Small model includes variables: NSS, LUTS, Age, surgical volume. *ASA* American Society of Anesthesiologists, *BMI* Body mass index, *LUTS* Lower urinary tract symptoms, *NSS* nerve sparing surgery

**Fig. 2:**
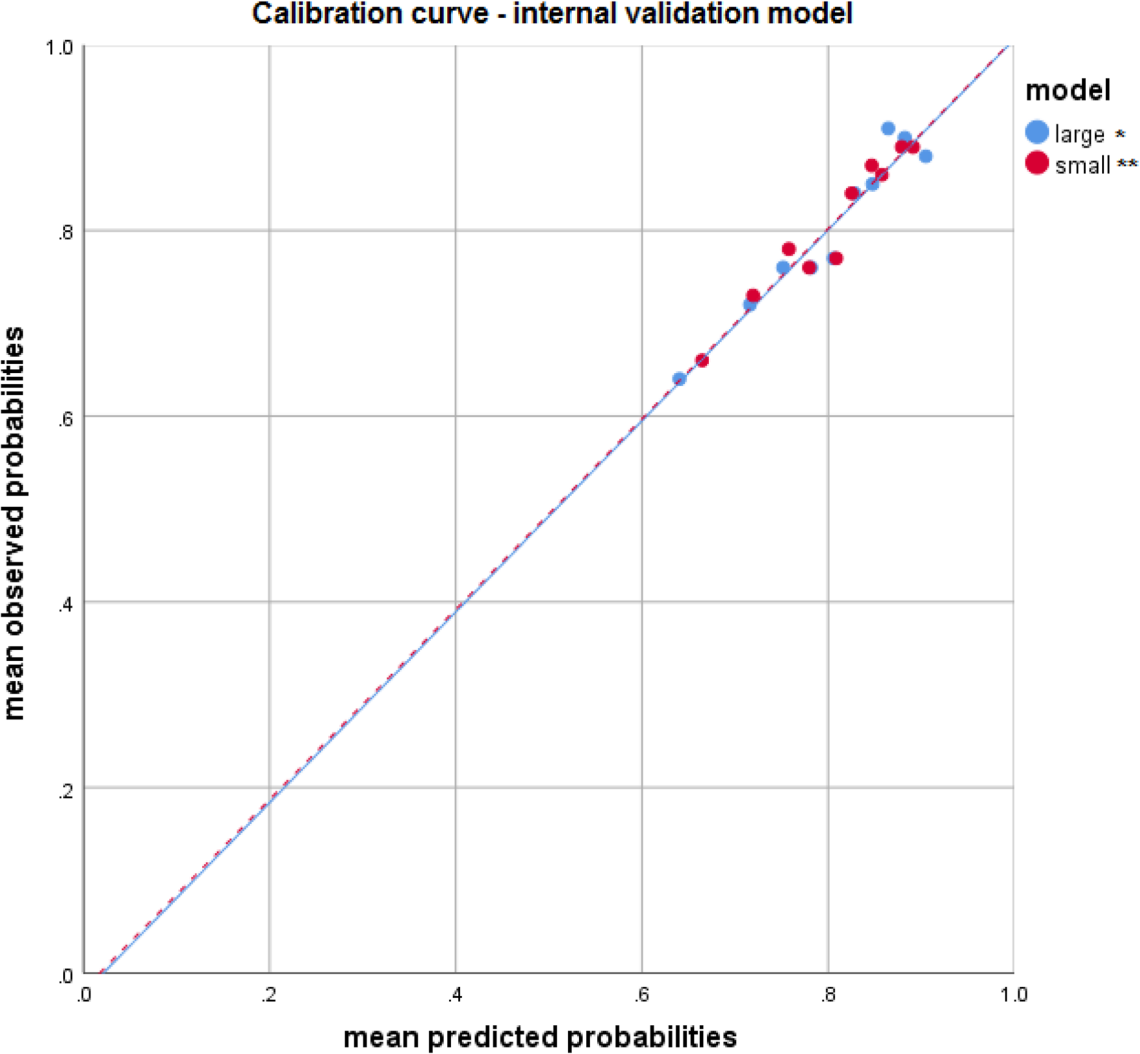
Calibration curve—internal validation model. We performed tenfold internal cross-validation. Internal validation of both models showed similar AUCs. The model performed well in predicting post-operative incontinence with poor discrimination and good calibration. *Large model includes variables: NSS, LUTS, Age, surgical volume, BMI, ASA, Prostate volume. **Small model includes variables: NSS, LUTS, Age, surgical volume. *ASA* American Society of Anesthesiologists, *BMI* Body mass index, *LUTS* Lower urinary tract symptoms, *NSS* nerve sparing surgery

The ROC curves for both models are presented in Fig. 1. The AUC of the large model was 0.65 (95% CI 0.61–0.68), while the AUC of the small model was 0.63 (95% CI 0.59–0.66), indicating that both models show a poor discrimination. The large model showed good calibration with a regression coefficient of 1.028 and intercept of − 0.022 (Fig. 2). Internal validation of both models showed similar AUCs: a mean AUC of 0.61 (95% CI range: 0.54–0.70) for the large model and a mean AUC 0.60 (CI 95% range: 0.53–0.67) for the small model.

**Fig. 3:**
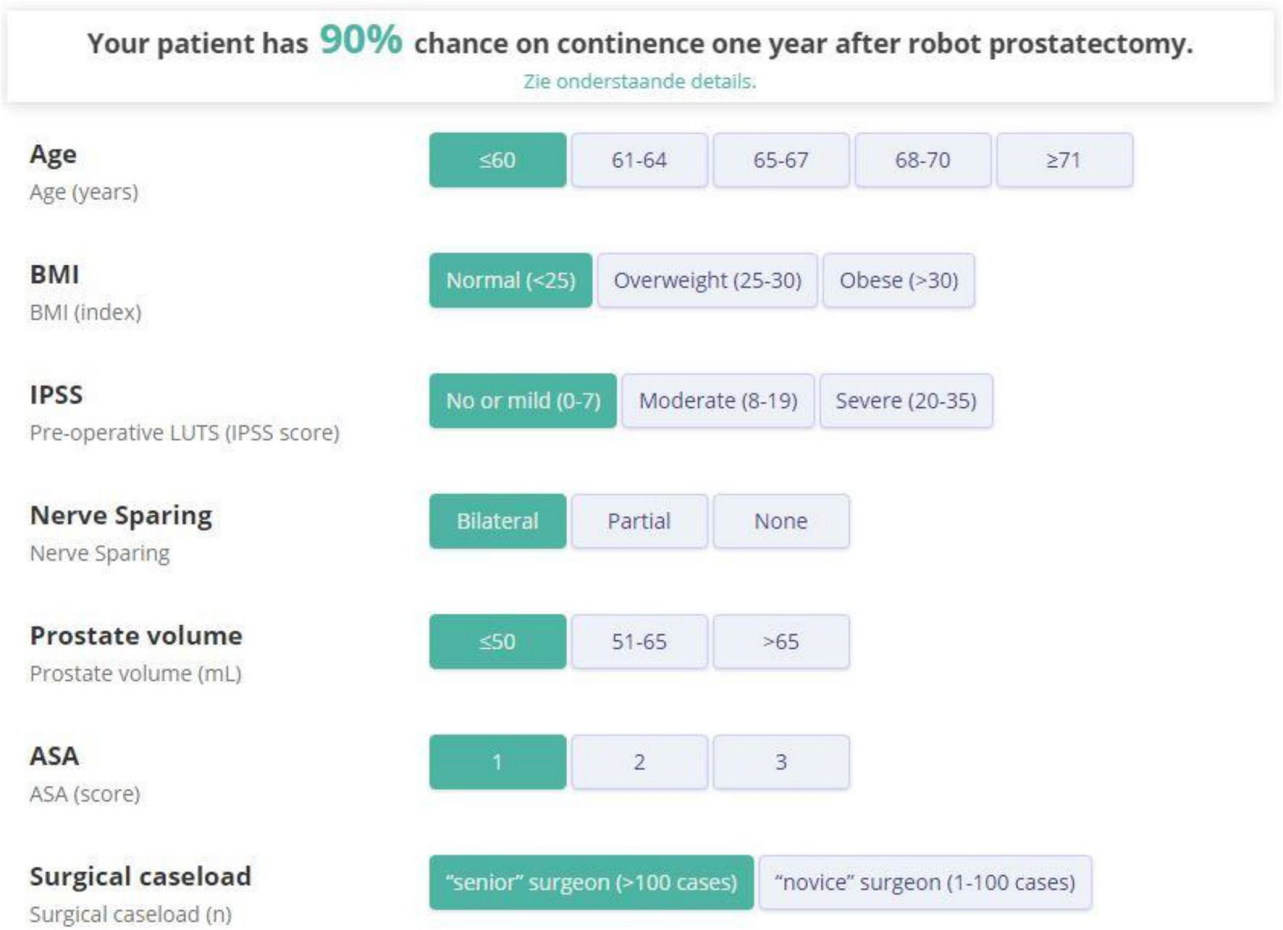
Online accessible continence prediction model. The patients’ characteristics can be enrolled in this online prediction model to assess the patients’ personal chance on continence. The regression coefficient and the intercept were extracted from the multivariable logistic regression model, which was used to assess the association between the pre-operative parameters and the primary outcome measure. The online prediction mode is available online and free accessible at www.collette.nl/calcu

The online prediction model, based on the multivariable regression model to predict 1 year post-operative continence is shown in Fig. 3 and is freely accessible online at www.collette.nl/calcu.

## Discussion

Postoperative urinary incontinence is one of the most bothersome side-effects of robot-assisted radical prostatectomy (RARP). If adequate prediction of PPI is possible, it may be a helpful tool in the counseling of patients in their choice of treatment after a diagnosis of prostate cancer. Also, patients at high risk for PPI could be withheld surgery, or if surgery remains mandatory, specific surgical techniques could be used to maximize functional outcomes [9]. This is one of the few studies that is aimed to develop an online prediction model for PPI after RARP that may be used by both clinicians and patients before treatment. Beside well-known risk factors for PPI as are described in the urological literature such as (older) age and the severity of lower urinary tract symptoms, this study used a set of less commonly used predictive factors for PPI such as the extent of nerve-sparing surgery and surgical experience.

The present study reports on a large consecutive cohort of patients with a relatively long median follow-up of 36 months (range 12–108) compared to similar studies on this subject. The continence rates were defined by patient reporting outcome measures (PROMS) using validated questionnaires that were handed over to patients at set and well-defined time point after surgery. Here, a properly used definition of urine continence was used as is recommended by the European Association of Urology, i.e. the use of zero pads or the use of only one safety pad per day. One of the major strengths of the present study is the high response rate of men returning the PROMS questionnaires (i.e., 85.2%), the large size of the study cohort (i.e., 1814 patients), and the important primary endpoint (i.e., PPI at one year after RALP).

Herein, a wide series of clinical, and surgical variables, i.e. older age, higher BMI, higher prostatic volume, higher ASA-score, the severity of LUTS, absence of nerve-sparing surgery and less surgical experience were all statistically significant risk factors for poor outcome on univariate analysis when PPI was evaluated (Table 2). In multivariable logistic regression analysis, we found pre-operative severity of LUTS, higher age, the extent of nerve sparing surgery and surgeon experience to be significant independent predictors of post-operative continence status (Table 3). ASA, BMI and prostate volume were not significantly associated with continence. Based on these data, an online prediction model for PPI status was developed. At internal validation, our online prediction model performed well in predicting PPI with poor discrimination and good calibration (Fig. 2). The online prediction mode is available online and free accessible at www.collette.nl/calcu.

There are only few studies predicting PPI using validated nomograms. AUC values ranged from 0.67 to 0.71. Nomograms have the highest accuracy and best discriminating characteristics among the various prediction tools, as they are constructed through multivariable models. By our knowledge, no online prediction model of PPI exists.

Matsushita et al. included clinical parameters such as patient age, BMI, ASA score and multiparametric magnetic resonance imaging (mpMRI) measured membranous urethral length (MUL) to develop a predictive model for PPI. In this retrospective analysis of data from 2849 patients undergoing RARP using multivariable logistic regression analysis, an intuitive nomogram was developed that could be used to counsel patients on their risk of PPI after radical prostatectomy. However, one of the backsides of this study is that surgical experience and LUTS were not assessed in their predictive model. Also, PROMs were not used in data gathering [6].

Based on a consecutive series of 1168 robot-assisted and open retropubic radical prostatectomies, Jeong et al. developed and validated nomograms to predict early, intermediate and late recovery from urinary incontinence after surgery using a multivariate model. Age at surgery, MUL, and robot-assisted radical prostatectomy were significant for recovery of incontinence at 1, 3, and 12 months. Saving the neurovascular bundle (NVB) and prostate volume were significant only for recovery of incontinence at 12 months. Severity of LUTS and surgical experience were not included into the model [10]. Also, only a part of patients underwent pre-operative mpMRI so could not benefit from this prediction model.

In a study on functional outcomes after nerve-sparing radical prostatectomy in 1311 patients, a nomogram was developed based on age, BMI, erectile function, surgery type (open or robotic) and extent of nerve sparing type. PROMs were filled in during outpatient clinic. Pre-operative LUTS and surgical experience were not assessed in the nomogram [11]. Patients with urinary incontinence consisted mainly of individuals who were impotent before RP, elderly and/or overweight. A nomogram was based on 209 patients' age-adjusted Charlson’s comorbidity index, Erectile Function, prostate volume, nerve-sparing status and 24-h urine loss at 1 month after RARP to predict 1-year PPI. The study is hampered by low level of statistical power due to a small sample group [12].

The present study may be hampered by several pitfalls. It may well be that certain predictors for PPI were not included in the present analysis where these were reported to have prognostic impact in previous studies. These variables may be previous transurethral resection of the prostate (TURP) prior to RARP, or the presence of specific anatomical features such as the presence of a (large) middle prostatic lobe or posterior prostatic lobe. Most importantly, the variables that can, at present, be evaluated on pre-operative mpMRI were not used in the present predictive model. This holds true for the MUL and the inter-levator distance (ILD) which have recently shown to have prognostic impact for PPI [6, 10]. However, at the time that the present cohort of patients underwent RARP, mpMRI was not yet performed as a routine diagnostic imaging modality in the clear majority of patients. Also, MUL is known to be not properly defined yet by radiologist and is therefore prone for high interobserver variability. At last, patients who underwent oncological treatment due to progression of disease such as by salvage radiation therapy to the prostatic fossa or hormonal medication were censored for the primary outcome of PPI at the time treatment was initiated. It might well be that the urinary continence rate might have improved further by continued follow-up, whereas this was censored at the time of last follow-up. On the other hand, most patients will not have started salvage oncological treatment within 1 year after RARP.

Due to the complexity and challenging nature of radical prostatectomy (RP), it is likely that both short- and long-term outcomes of urinary incontinence strongly depend on the cumulative number of cases performed by the surgeon as well as by the hospital. It has been a subject of long debate to what extent this influences continence outcomes and what the threshold is for the number of cases that need to be treated to gain optimal outcomes. Trinh et al. performed a systematic review in which the association between hospital and surgeon volume and perioperative, oncologic, and functional outcomes after RP were assessed. From 45 studies reporting on this subject retrospectively, undeniable evidence suggests that increasing surgical volume improves outcomes. A similar study was performed by Wilt et al. In their systematic review, the association between hospital and surgeon volume, and patient outcomes after RP were assessed. The rate of late urinary complications was 2.4% lower (95% CI − 5, − 0.1) and the rate of long-term incontinence was 1.2% lower (95% CI − 2.5, − 0.1) for each 10 additional RP performed by the surgeon annually. So, higher provider volumes of surgery are associated with better outcomes after RP and an argument to implement this variable in future models predicting PPI after RARP [13, 14].

## Conclusion

Using consecutive data from a large, well-defined cohort of patients who underwent RARP in a single robotic center, a predictive model for PPI at one year postoperatively was developed. The study reported a high response rate (85.2%), a long median follow-up time (36 months (range 12–108), and used validated PROMS to define the primary endpoint of PPI. On univariate analysis, all variables were associated with PPI. In multivariable logistic regression analysis, severity of LUTS, higher age, extent of nerve sparing surgery and surgeons experience proved to be independent significant predictors of PPI. ASA, BMI and prostate volume were not significantly associated to PPI. An intuitive online prediction model to predict PPI was build which includes the independent predictors for PPI one year after RARP. At internal validation, our online prediction model performed well in predicting post-operative incontinence with poor discrimination and good calibration.

## Electronic supplementary material

Below is the link to the electronic supplementary material.

Supplementary file1 (DOCX 35 kb)
