# Supplementary material for: Patient reported outcome measures concerning urinary incontinence after robot assisted radical prostatectomy: development and validation of an online prediction model using clinical parameters, lower urinary tract symptoms and surgical experience
Source: J Robot Surg. 2020 Sep 15;15(4):593–602. doi: 10.1007/s11701-020-01145-9 (PMC8295126; doi:10.1007/s11701-020-01145-9)

**Optional Online supplement Figures**

**Figure**: Kaplan–Meier curve for the post-operative time to continence


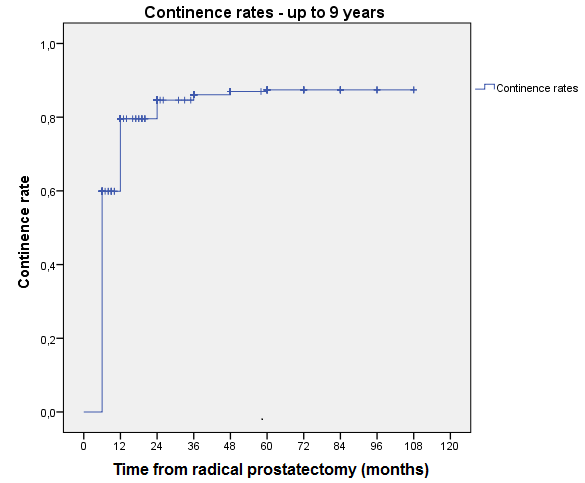

Supplement: Supplementary file 1 — Supplementary file1 (DOCX 35 kb) [file 11701_2020_1145_MOESM1_ESM.docx]
